# Patients’ dissatisfaction with multifocal intraocular lenses managed by exchange with other multifocal lenses of different optical profiles

**DOI:** 10.1186/s40662-022-00280-8

**Published:** 2022-03-01

**Authors:** Olena Al-Shymali, Colm McAlinden, Jorge L. Alio del Barrio, Mario Canto-Cerdan, Jorge L. Alio

**Affiliations:** 1 Research & Development Department VISSUM Innovation and Department of Cornea, Cataract and Refractive Surgery, VISSUM Corporation, Alicante, Spain; 2 Department of Ophthalmology, Singleton Hospital, Swansea Bay University Health Board, Swansea, UK; 3 Division of Ophthalmology, School of Medicine, VISSUM Alicante, Universidad Miguel Hernández, Calle Cabañal 1, 03016 Alicante, Spain; 4 Cornea, Cataract and Refractive Surgery Department, VISSUM Corporation, Alicante, Spain

## Abstract

**Background:**

The aim of the study was to evaluate the outcomes of dissatisfied patients reporting poor visual quality following implantation of multifocal intraocular lenses (MF-IOLs), managed by IOL exchange with another multifocal optical profile.

**Methods:**

This is a retrospective series of cases. MF-IOL exchange was done in 15 dissatisfied patients (30 eyes) with the perception of poor visual quality for far distance affected by neuroadaptation failure. Patients underwent a bilateral exchange of a MF-IOL with another MF-IOL of a different optical profile. Visual outcomes and complications were analyzed. Questionnaires including Quality of Vision (QoV), Visual Function Index-14 (VF-14) and its Rasch-revised version (VF-8R) and a satisfaction questionnaire were also used for outcome evaluation.

**Results:**

The mean elapsed time from implantation to explantation-reimplantation was 11.8 months. The QoV scores improved significantly across all the three subscales. Visual function improved with a change in VF-14 score from 60.41 ± 24.81 to 90.16 ± 10.91 (*P* < 0.001). The VF-8R score improved as well. The uncorrected distance visual acuity improved from 0.24 to 0.12 logMAR after exchange (*P* < 0.001) and corrected distance visual acuity improved from 0.15 to 0.04 logMAR (*P* < 0.001). Safety and efficacy indexes reached 1.46 and 1.16, respectively. Concerning patients’ satisfaction following MF-IOL exchange, 80% of the patients reported they would have the MF-IOL reimplantation procedure again.

**Conclusions:**

Patient dissatisfaction with neuroadaptation failure following MF-IOL implantation can be managed in 80% of our cases by MF-IOL exchange with a different MF-IOL optical profile.

## Background

Multifocal intraocular lenses (MF-IOLs) are used today to provide an adequate visual performance for far and near in pseudophakic patients. However, to accomplish their goal, they require a process of neuroadaptation that in some cases may fail, resulting in patients’ dissatisfaction, which eventually in severe cases may require the explantation of the MF-IOL.

Patient dissatisfaction with MF-IOLs is mainly due to subjective inadequate visual outcome, abnormal photic phenomena, dysphotopsia and/or subjective perception of poor quality of vision (QoV), especially for far distance, with no solid underlying organic reason such as posterior capsule opacification (PCO), dry eye, retinal disease, residual refractive error or other comorbidities [1–4]. Such symptoms constitute the process of neuroadaptation failure. The study of neuroadaptation to the complex image provided by MF-IOLs has been the subject of investigations that have demonstrated the involvement in this process of cortical regions dedicated to attention, learning and cognitive control [5, 6]. When a MF-IOL is implanted, a form of long-term adaptation occurs, where some brain regions are activated with time compared to the early postoperative period [6]. However, sometimes such neuroadaptation fails, and patients become unable to tolerate the unpleasant subjective visual phenomena associated with the implanted MF-IOL. Neuroadaptation to MF-IOLs seems to happen smoother and faster when MF-IOLs are implanted in both eyes within a short time frame [7].

Several authors have reported the outcomes of the management of patient dissatisfaction with MF-IOL exchange with a monofocal IOL[8–15]. In this alternative solution, the patient loses the ability to simultaneously focus for far and near, which in the first place was the aim of the patient to be accomplished by choosing the MF-IOL. However, to the best of our knowledge, no study has previously evaluated the alternative of MF-IOL exchange by another MF-IOL of a different optical profile, an option that would keep the advantage of spectacle near vision independence, if successful.

The aim of this study was to assess the outcomes of this alternative therapeutic approach for the management of patient dissatisfaction related to the perception of poor distance visual quality following MF-IOL implantation.

## Methods

This study was conducted to evaluate retrospectively the visual and clinical outcomes as well as the satisfaction of patients who had explantation of a multifocal IOL (MF1) followed by the implantation of another multifocal IOL (MF2) of a different optical profile. These patients were significantly dissatisfied following MF-IOL surgery due to poor perception of far vision quality. Institutional Ethical Board Committee approval was obtained for the purpose of this investigation. All patients signed an adequate informed consent, and the study was conducted in accordance with the tenets of Declaration of Helsinki (64th WMA General Assembly, Fortaleza, Brazil, October 2013).

### Patient selection

This study included 30 eyes of 15 operated patients (9 females and 6 males). Patients were bilaterally implanted with a MF-IOL, either refractive, diffractive or extended depth of focus (EDOF), who developed neuroadaptation failure as mentioned above, reporting significant dissatisfaction [7]. They were informed of the potential benefits and limitations in terms of near vision performance of the exchange of the MF-IOL either by a monofocal one or by another MF-IOL of a different optical profile depending on the hypothesis followed by the authors of this study about the possibilities of keeping the multifocal advantages. Cases that selected the monofocal option are subjects of a different investigation.

For those who chose to exchange with a different MF-IOL, the selection of the new IOL was performed as follows: Cases implanted with any type of diffractive technology were explanted and exchanged by a refractive optical design. Those implanted with a refractive multifocal optic were exchanged with a diffractive MF-IOL. EDOF lenses were exchanged with either a diffractive or refractive MF-IOL. Such decision was made under the hypothesis that different optical designs activate and follow different neuroadaptation mechanisms. IOL explantation was decided in general after 6 months of significant patient complains related to neuroadaptation failure. In Table 1, we show different models of MF-IOLs implanted in the patients selected for this investigation and the MF-IOLs that were used for the purpose of the exchange. Table 2 shows the physical design description of the MF-IOLs used in this study. Before the implantation of MF2, a new biometry was done and the power of the new MF-IOL was calculated as usual for pseudophakic eyes. All patients presented with high-order aberrations under 0.3 µm. Angle Kappa was evaluated, and no patient had more than 1 mm of angle Kappa.

**Table 1:** Patients’ data

Patient No.	Gender	Age (years)	Eye	MF1	MF2	T(m)	Repeat surgery
1	M	63	R	Lentis Mplus LS-313 MF 15	AT LISA tri 839MP	3	Yes
			L	Lentis Mplus LS-313 MF 30	AT LISA tri 839MP	3	
2	M	58	R	AcrySof IQ ReSTOR SN6AD1	Lentis Mplus LS-313 MF30	3	Yes
			L	AcrySof IQ ReSTOR SN6AD1	Lentis Mplus LS-313 MF15	3	
3	M	47	R	Mini Well Ready	Lentis Mplus LS-313 MF 15	9	Yes
			L	Mini Well Ready	Lentis Mplus LS-313 MF 30	9	
4	F	67	R	AcrySof IQ PanOptix TFNT00	Lentis Mplus LS-313 MF 30	7	Yes
			L	AcrySof IQ PanOptix TFNT00	Lentis Mplus LS-313 MF15	3	
5	F	54	R	Lentis Mplus LS-313 MF 30	AT LISA tri 839MP	26	Yes
			L	Lentis Mplus LS-313 MF 30	AT LISA tri 839MP	26	
6	F	55	R	M-flex 630-F	AT LISA tri 839MP	12	Yes
			L	M-flex 630-F	AT LISA tri 839MP	12	
7	F	62	R	MiniWell Ready	AT LISA tri 839MP	3	Yes
			L	MiniWell Ready	AT LISA tri 839MP	3	
8	M	54	R	MiniWell Ready	Lentis Mplus LS-313 MF 15	3	Yes
			L	MiniWell Ready	Lentis Mplus LS-313 MF 15	3	
9	F	62	R	Lentis Mplus LS-313 MF 30	AT LISA 809 M	3	No
			L	Lentis Mplus LS-313 MF 30	Acri Lisa 366D	3	
10	F	70	R	AcrySof IQ ReSTOR SN6AD3	Lentis Mplus LS-313 MF 30	89	Yes
			L	AcrySof IQ ReSTOR SN6AD3	Lentis Mplus LS-313 MF 30	89	
11	F	61	R	Lentis Mplus LS 313 MF 15	AcrySof IQ PanOptix TFNT00	5	No
			L	Lentis Mplus LS-313 MF 30	AcrySof IQ PanOptix TFNT00	4	
12	F	57	R	Precizon Presbyopic	AT LISA tri 839MP	7	Yes
			L	Precizon Presbyopic	AT LISA tri 839MP	7	
13	M	50	R	Precizon Presbyopic	FineVision Pod F	3	Yes
			L	Precizon Presbyopic	FineVision Pod F	3	
14	F	47	R	Acunex Vario	AT LISA tri 839MP	3	Yes
			L	Lentis Mplus LS 313 MF 15	AT LISA tri 839MP	3	
15	M	57	R	Precizon Presbyopic	Intensity SL HP	3	No
			L	Precizon Presbyopic	Intensity SL HP	3	

*MF1* = the first multifocal intraocular lens; *MF2* = the second multifocal intraocular lens; *T(m)* = time between implantation and explantation in months; *M* = male; *F* = female; *R* = right; *L* = left

**Table 2:** Multifocal intraocular lenses physical design description

IOL model	IOL type	IOL physical design description
Lentis Mplus LS-313 MF (Oculentis)	Refractive	Optic size: 6.0 mm Overall size: 11.0 mm Haptic style: plate with 0° angulation
AT LISA tri 839MP (Zeiss)	Diffractive	Optic size: 6.0 mm Overall size: 11.0 mm Haptic style: plate with 0° angulation
AcrySof IQ ReSTOR SN6AD1/D3 (Alcon)	Diffractive	Optic size: 6.0 mm Overall size: 13.0 mm Haptic style: STABLEFORCE® Modified-L Haptics with 0° angulation Diffractive steps: 9
Mini Well Ready (Sifi Medtech)	EDOF	Optic size: 6.0 mm Overall size: 10.75 mm Haptic style: Four haptics in closed double–C loop with 5° haptic angulation
AcrySof IQ PanOptix TFNT00 (Alcon)	Diffractive	Optic size: 6.0 mm Overall size: 13.0 mm Haptic style: STABLEFORCE™ Modified-L Haptics with 0° angulation
AT Lisa 809 M (Zeiss)	Diffractive	Optic size: 6.0 mm Overall size: 11.0 mm Haptic style: plate with 0° angulation
FineVision Pod F (PhysIOL)	Diffractive	Optic size: 6.0 mm Overall size: 11.4 mm Haptic style: double C-loop haptics with 5° haptic angulation
M-flex 630-F (Rayner)	Refractive	Optic size: 6.25 mm Overall size: 12.5 mm Haptic style: Closed loop with anti-vaulting haptic with 0° haptic angulation
Precizon Presbyopic (Ophtec BV)	Refractive	Optic size: 6.0 mm Overall size: 12.5 mm Haptic style: open modified-C loops with offset-shaped haptics with 0° angulation
Acri Lisa 366D (Zeiss)	Diffractive	Optic size: 6.0 mm Overall size: 11.0 mm Haptic style: plate with 0° angulation
Acunex Vario (Teleon Surgical)	EDOF	Optic size: 6.0 mm Overall size: 12.5 mm Haptic style: C-loop haptics with 0° angulation
Intensity SL HP (Hanita Lenses)	Diffractive	Optic size: 6.0 mm Overall size: 13.0 mm Haptic style: C-loop haptics with 5° angulation

*IOL* = intraocular lens; *EDOF* = extended depth of focus

All explantations and reimplantations were done by the same surgeon (JLA) at VISSUM Ophthalmological Institute, Miranza Group (Alicante, Spain), Miguel Hernandez University.

### Definition and identification of neuroadaptation failure

Neuroadaptation is a process in which the nervous system adjusts and adapts to changes in neural inputs, and thus it is when the brain learns how to “correct” the abnormal or atypical image so it resembles an acceptable one [7]. Neuroadaptation is an acquired learning process, so our brain gets used to regulating the visual input according to what it already knows as the disturbances and aberrations. MF-IOLs, due to their design, lead to a multifocal image which is rarely presented in the human visual system. Implanting a MF-IOL creates a different distribution of light and creates a superimposition of images that makes the brain accept different images located at different focal distances [7]. Consequently, the brain may find it more difficult to percept a well-detailed image. If the brain fails to finally adapt and cannot sense of a well-detailed image, neuroadaptation failure may appear. In our study, we considered that patients had neuroadaptation failure when they reported poor QoV, sometimes associated to a bilateral decrease of the expected best corrected visual acuity. This failure causes patient dissatisfaction and is to be differentiated from the dissatisfaction provided by an inadequate spectacle independent near visual outcome.

Residual ametropia for far distance vision may interfere significantly with subjective satisfaction following MF-IOL surgery. For this reason, a trial with the prescription of glasses was performed in all the cases of the study prior to consider MF-IOL explantation in order to rule out the potential participation of residual refractive error as a cause of the dissatisfaction [16]. Wearing glasses was prescribed for a minimum of one month when a spherical equivalent of more than 1 diopter (D) was present in either eye [16]. All cases included in this study were still not satisfied with the far vision outcome even with the use of glasses for the residual refractive error. No other organic reason was found to justify the inadequate visual perception in such cases. Consequently, neuroadaptation failure diagnosis was confirmed in all the cases included in this investigation.

### Surgical technique

The MF-IOL was explanted from the capsular bag, frequently assisted by 2 or 3 radial cuts in the anterior capsular rim. Abundant cohesive viscoelastic was used to open the capsular bag, dissecting the lens inside it, releasing the adhesions, and finally extruding it into the anterior chamber. The MF-IOL was extracted from the eye through a 3-mm incision made in the positive meridian of the corneal topography once it was bisected in two or cut radially and pulled out from the eye by rotation in the incision [17, 18]. The new MF-IOL was always implanted in an emptied capsular bag. None of the patients had a YAG-capsulotomy prior to the IOL exchange.

### Main outcome measures

Patient’s outcomes were evaluated following 3 months of postoperative evolution, as follows:

#### Visual outcomes

Uncorrected and best corrected visual acuities for far at 5 m distance and near at 40 cm distance were measured. Refractive outcomes were evaluated as well (Table 3).

**Table 3:** Refractive and visual outcomes following the first multifocal intraocular lens (MF1) and the second multifocal intraocular lens (MF2) implantation

	MF1		MF2		*P* value
	Mean ± SD	Range	Mean ± SD	Range	
UDVA (logMAR)	0.24 ± 0.61	1.7–0.0	0.12 ± 0.77	0.44–0.00	< 0.001
CDVA (logMAR)	0.15 ± 0.57	1.7–0.0	0.04 ± 0.98	0.26–0.00	< 0.001
Sph (D)	0.21 ± 0.67	− 1.00–1.25	0.38 ± 0.44	− 0.50–1.50	0.111
Cyl (D)	− 0.35 ± 0.49	− 1.25–1.25	− 0.68 ± 0.45	− 1.50–0.00	0.010
SE (D)	0.03 ± 0.59	− 1.00–1.00	0.04 ± 0.43	− 0.75–1.00	0.884
UNVA (logMAR)	0.31 ± 0.66	1.4–0.0	0.28 ± 0.68	0.62–0.00	0.076
DCNVA (logMAR)	0.26 ± 0.56	1.4–0.0	0.20 ± 0.62	0.52–0.00	0.235

*UDVA* = uncorrected distance visual acuity; *CDVA* = corrected distance visual acuity; *Sph* = sphere; *Cyl* = cylinder; *SE* = spherical equivalent; *UNVA* = uncorrected near visual acuity; *DCNVA* = distance-corrected near visual acuity; *SD* = standard deviation

#### QoV evaluation by questionnaire Rasch scores

The QoV questionnaire was used 3 months after each MF-IOL implantation (Table 4) [19]. The patients of the study rated 10 visual symptoms on the basis of their frequency, severity and bothersomeness. Glare, halos, starbursts, hazy vision, blurred vison, distortion, double or multiple images, fluctuations in vison, focusing difficulties, and difficulties in judging distance or depth perception were assessed. Analysis was applied to raw data and the scores were converted to final 0–100 Rasch-scale with lower scores indicating better QoV [20].

**Table 4:** Quality of Vision questionnaire Rasch score

	Preoperative score (mean ± SD)	Postoperative score (mean ± SD)	*P* value
Frequency	61.27 ± 16.42	35.87 ± 29.40	0.012
Severity	54.20 ± 17.83	34.20 ± 32.26	0.042
Bothersome	59.67 ± 18.13	32.33 ± 35.97	0.019

Scores of the patients before the exchange of a multifocal IOL and after the exchange to another multifocal IOL. The greater the score (maximum 100), the worse is the quality of vision. *SD* = standard deviation

#### Subjective Visual Function Index-14 (VF-14) evaluation

The subjective VF-14 questionnaire was used 3 months after implantation of each of the MF-IOLs (Table 5). This questionnaire includes 14 questions on the difficulties that patients encounter in their daily life activities. The total score was calculated by a previously described method [21]. To study the visual function in daily activities at different distances, we divided the questions into 3 groups: six questions for far vision, three questions for intermediate vision and five questions for near vision. Due to concerns raised over the scoring of the original VF-14, we have also performed Rasch-analysis (using WINSTEPS, Version 3.93.2, Chicago, IL) to score the 8 items of the refined version. This version is known as the VF-8R [22].

**Table 5:** Visual Function Index-14 questionnaire score

	Preoperative score (mean ± SD)	Postoperative score (mean ± SD)	*P* value
Total score	60.41 ± 24.81	90.16 ± 10.91	< 0.001
Far distance	61.55 ± 22.98	89.70 ± 13.03	< 0.001
Intermediate distance	68.89 ± 26.63	97.50 ± 7.01	0.001
Near distance	58.00 ± 30.17	86.00 ± 14.78	0.004

Mean scores of the patients before the exchange of a multifocal IOL and after the exchange to another multifocal IOL. The greater the score (maximum 100), the better is the visual function. *SD *= standard deviation

#### Patient satisfaction evaluation

Patients were asked about their overall satisfaction with their near, intermediate, and far vision, spectacle independency for these distances and if the patient would repeat the surgery again (Table 6).

**Table 6:** Percentage of patients answering 4 questions regarding their overall satisfaction with their vision for different distances, their willingness to repeat the surgery, their spectacle independency, and the frequency of spectacle use, all with the first multifocal intraocular lens (MF1) versus the second multifocal intraocular lens (MF2)

	What was your overall satisfaction with the vision?					
	With MF1			With MF2		
	Far (%)	Intermediate (%)	Near (%)	Far (%)	Intermediate (%)	Near (%)
Very good	6.67	0.00	0.00	20.00	46.67	33.33
Good	13.33	26.67	26.67	60.00	26.67	33.33
Average	40.00	40.00	33.33	0.00	6.67	13.33
Bad	0.00	6.67	6.67	13.33	13.33	20.00
Very bad	40.00	26.67	33.33	6.67	6.67	0.00
	Would you repeat the surgery?					
	With MF1 (%)			With MF2 (%)		
Yes	13.33			80.00		
No	86.67			20.00		
	Were you spectacle independent?					
	With MF1			With MF2		
	Far (%)	Intermediate (%)	Near (%)	Far (%)	Intermediate (%)	Near (%)
Yes	86.67	80.00	60.00	93.33	93.33	73.33
No	13.33	20.00	40.00	6.67	6.67	26.67
	How often you used spectacles?					
	With MF1			With MF2		
	Far (%)	Intermediate (%)	Near (%)	Far (%)	Intermediate (%)	Near (%)
Never	93.33	86.67	66.67	100.00	100.00	66.67
Almost never	6.67	6.67	6.67	0.00	0.00	6.67
Sometimes	0.00	0.00	6.67	0.00	0.00	0.00
Almost always	0.00	0.00	6.67	0.00	0.00	0.00
Always	0.00	6.67	13.33	0.00	0.00	26.67

### Statistical analysis

SPSS for windows (IBM SPSS Corporation, version 18, USA) and Student’s t-test were used. The data analyzed are expressed as the mean ± standard deviation (SD) and a *P* value of less than 0.05 was considered statistically significant.

## Results

The present study included patients affected by significant dissatisfaction related to neuroadaptation failure following implantation of different types of MF-IOLs (Table 1). MF-IOL exchange by another MF-IOL with a different optical profile was decided according to the previously postulated hypothesis. The mean age of the patients was 58.0 ± 6.7 years (range: 47 to 70 years). The mean time between the two surgeries was 11.8 ± 21.8 months with a median of 3 months. The type of MF-IOL explanted (MF1) and the type of lens with which the patient was reimplanted (MF2) are summarized in Tables 1 and 2, while the visual outcomes are shown in Table 3.

As shown in Table 3, the mean uncorrected distance visual acuity (UDVA) increased significantly from 0.24 logMAR with MF1 to 0.12 logMAR with MF2 (*P* < 0.001). Also, corrected distance visual acuity (CDVA) improved from 0.15 to 0.04 logMAR (*P* = 0.000) and the binocular distance vision from 0.05 to − 0.01 logMAR (*P* = 0.002). The spherical equivalent refraction changed insignificantly from 0.03 to 0.04 D (*P* = 0.884). Both uncorrected near visual acuity (UNVA) and distance-corrected near visual acuity (DCNVA) had no significant change, from 0.31 to 0.28 logMAR (*P* = 0.076) and from 0.26 to 0.20 logMAR (*P* = 0.235), respectively. Safety and efficacy indexes reached 1.46 and 1.16 respectively. None of the eyes lost 1 or more lines of vision and 27% gained 2 or more lines. Of the 30 eyes, 80% were within ± 0.5 D and 100% were within ± 1.0 D. Only 7 eyes had a follow-up time of 1 year where the mean UDVA was 0.2 logMAR and mean CDVA was 0.04 logMAR.

A correlation analysis was performed using Spearman’s Rho statistic and there were no statistically significant correlations (*P* > 0.05) between the lens exchange time (time between implantation of MF1 and implantation of MF2) and the QoV frequency, QoV severity and QoV bothersome values. There were also no statistically significant correlations (*P* > 0.05) between the lens exchange time and the values of UDVA, CDVA, sphere, cylinder, spherical equivalent, UNVA, efficacy and safety.

### Subjective QoV

All the three QoV subscales improved significantly following implantation of MF2 (Table 4).

### Visual function index-14

All the VF-14 scores increased significantly after the exchange of MF1 to MF2, indicating a significant improvement in visual function (Table 5). The Rasch version of the VF-14, the VF-8R, showed that the mean (± SD) pre-exchange score was − 1.00 ± 1.91 and improved to − 3.32 ± 1.42 (*P* = 0.001) post-exchange, indicating an improvement.

### Patient satisfaction

We observed an increase in the percentage of satisfied patients after the MF-IOL exchange (Table 6). Nonetheless, 20% of the patients had bad or very bad satisfaction with their vision for far, intermediate or near vision with MF2. One of the patients (patient 2), had a bad satisfaction with his near vision postoperative with focusing difficulties leading to the necessity of wearing glasses for reading. Another patient (patient 7) answered that he had a bad satisfaction with his postoperative vision for all three distances. Despite very good UDVA and UNVA, he complained of blurred vision, glare and halos and sometimes double vision. The next patient (patient 8) complained of bad satisfaction for far and intermediate vision with MF2. Although the last patient (patient 9) had a better UDVA with MF2 comparing to MF1, he answered that he had a very bad satisfaction for far and intermediate vision and bad satisfaction for near vision. Both patients had various subjective symptoms such as photic phenomena, blurred vision, and fluctuation. Perhaps for patient 9, these subjective symptoms could be related to the moderate decentration of MF2 in both eyes postoperatively. These patients will require further approach such as a final exchange of the MF-IOL to a monofocal IOL which will be the subject of another manuscript.

When patients were asked if they would repeat the surgery, 13.3% answered yes with MF1 versus 80% with MF2. The patients (two patients) that answered yes to MF1 were happier with their vision compared to the one they had with the cataract, but their expectations from MF1 were not met. One patient was not satisfied with his far vision with MF1 and then became totally satisfied with MF2. Another patient despite good vision, suffered from starbursts and distortion with MF1 that disappeared with MF2. The results of spectacle independency and frequency of spectacle use are also shown in Table 6.

### Complications

Intraoperative complications: zonular dehiscence happened in both eyes of one patient (patient 10) that had the MF1 for 89 months, followed by a successful MF2 implantation in both eyes after the implantation of a capsular tension ring. Postoperative complications included: PCO requiring a laser posterior capsulotomy in two eyes. Two eyes of a patient showed a moderate decentration of the reimplanted MF2 IOL (a diffractive model), with no impact in the visual outcome.

## Discussion

MF-IOL explantation is happening more frequently now due to the increased use of this technology to correct pseudophakic presbyopia. With the development of new MF-IOL technologies, the indications for this procedure have also changed over time [23]. In 2012, our group analyzed the reasons for pseudophakic IOL explantation [9]. We concluded that the main causes for explantation were IOL dislocation/decentration, followed by incorrect lens power and IOL opacification. Few cases in their series (6.2%) had MF-IOLs explanted due to neuroadaptation failure. But the number of MF-IOLs implanted and other so called “Premium IOLs” has increased enormously along the last 10 years and explantations related to neuroadaptation failure are considered today to occur more frequently.

MF-IOLs are delicate in their optical performance and are more prone to create problems in the implanted eye that interfere with the neuroadaptation process, which is necessary for the clinical success of these lenses. Some studies have dealt with different aspects of pseudophakic IOL explantations [9, 13, 15, 24], but only a few of them investigated MF-IOL explantations [8, 10, 12, 14, 25]. Further, to the best of our knowledge there has been no study addressing exchanging one MF-IOL with a different MF-IOL model to keep on the advantages of near vision and spectacle independency. This study offers an outlook on MF-IOL explantation due to neuroadaptation failure and the outcomes following the exchange with another MF-IOL. MF2 was selected based on a MF-IOL with an equivalent visual performance to MF1 but using a different optical basis and technology. For example, a refractive MF-IOL was exchanged with a diffractive or EDOF MF-IOL, and conversely if the initial IOL optic was diffractive or EDOF.

The results of this study initially show for the first time the therapeutic value of the possibility of exchanging MF-IOLs with another MF-IOL with a different optical profile, demonstrating success in patients that were dissatisfied with their current QoV but were not willing to give up on the near vision advantages of MF-IOLs. Our results show that, in such cases, patients’ QoV as well as their visual function improved significantly after the exchange of MF1 to MF2. The overall satisfaction with the vision in all distances also significantly increased after the exchange. Yet, 20% of patients answered they have bad or very bad satisfaction with their vision for far, intermediate, or near with MF2. It is difficult to pinpoint the exact cause and to explain this dissatisfaction. A larger series of cases is needed for more accurate analysis. However, according to this series, the exchange of one MF-IOL to another has a failure rate of 20%, where these cases persisted with neuroadaptation failure even after the new MF-IOL design was implanted. Moreover, when patients were asked if they would repeat the surgery, 80% answered yes with MF2. Spectacle independency increased from 86.67 to 93.33% for far, from 80 to 93.33% for intermediate and from 60 to 73.33% for near vision. UDVA and CDVA increased significantly in addition to an improvement in both UNVA and DCNVA, while the refraction changed insignificantly. Although the explantation of an IOL is potentially associated with surgical risks, we observed zonular dehiscence in 2 eyes with no other complication during the follow up period of the study. An earlier decision of MF-IOL exchange could probably reduce the frequency of such complications as early explantation is easier.

Kamiya et al. in a case series of 50 eyes, reported the causes for MF-IOL explantations (42 diffractive and 8 refractive), including: decreased contrast sensitivity, followed by photic phenomena, unknown origin including neuroadaptation failure, incorrect IOL power, preoperative excessive expectations, IOL decentration/dislocation, and anisometropia [10]. Only 5 of the eyes, afterwards, were implanted with multifocal IOLs, and that was due to an incorrect IOL power calculation in the first place. Both Galor et al. and Kim et al. found out that blurred vision and photic phenomena were the most common indications for MF-IOL explantation in their series, where all cases afterwards were implanted with monofocal IOLs [8, 12]. Our report is focusing on and studying the failure of neuroadaptation as a main cause for dissatisfaction after MF-IOL implantation.

Some authors suggested that the best option for dissatisfied patients with MF-IOLs is the exchange to a monofocal IOL [13]. Nowadays, this may not be the best option considering high patient expectation and desire for spectacle independence at all distances despite the risk of persistent negative visual symptoms and its consequent dissatisfaction induced by MF-IOLs, in a patient already dissatisfied under similar circumstances. Thus, we suggest the feasibility of MF-IOL exchange with another MF model while preserving the near vision advantages of MF-IOLs and improving the undesired photic phenomena or dissatisfaction due to failure of neuroadaptation. However, MF-IOL exchange into another MF-IOL might be a complex procedure that can be influenced by various challenges, such as capsular bag retraction or disruption, zonular dehiscence, and posterior capsular rupture. In some cases, with dissatisfaction due to neuroadaptation failure that develop PCO, it is important to postpone the YAG-capsulotomy if the final decision will be MF-IOL exchange. This will guarantee a less risky exchange surgery. For the same reason, it is pivotal to maintain the capsular bag during the surgery itself while removing the MF-IOL to place the second MF-IOL into the bag.

A similar situation happened to one of the patients in this series. One eye of this patient, as seen in Table 2, had a very low CDVA of 1.7 logMAR before the exchange. This patient was implanted with MF1 in another center, being unhappy from the beginning and experienced a lot of halos, starbursts, and glare. He came to our center for a second opinion and since he presented with severe symptoms of neuroadaptation failure since the original implantation, we proposed a MF-IOL exchange despite some early PCO present. While the patient was deciding on whether to go with the surgery or not, he developed further PCO in this eye, being the reason for such low preoperative vision in this eye. However, as previously discussed, we did not perform a YAG-capsulotomy, but instead we performed a MF-IOL exchange surgery with posterior capsule polishing, aiming for a YAG capsulotomy along the postoperative if necessary.

Depending on the patients’ needs and expectations, different approaches can be used. If the patient is unhappy with the MF-IOLs, monovision or mini-monovision with monofocal IOLs can be used [26]. The amount of residual refractive error in the reading eye can reach up to − 2.50 D or more in traditional monovision [26]. Such anisometropia can lead to neuroadaptation failure as well. Blended implantation of MF-IOLs is also an option where bilaterally diffractive or refractive MF-IOLs are implanted but with different near add powers [27–29]. In fact, some of our patients, such as patients 1 and 11, were implanted with the refractive MF-IOL Lentis Mplus with different near addition but still developed neuroadaptation failure. A blended vision option with EDOF IOLs was described, where an EDOF lens was implanted in one eye, and a MF-IOL was implanted in the second eye. The authors compared this group to a group of patients where EDOF IOLs were implanted bilaterally and better results for near visual acuity were demonstrated in the mixed group [30]. A similar study was done where patients were implanted with EDOF IOL in one eye and a monofocal IOL in the other eye and compared to a group of patients bilaterally implanted with EDOF IOLs. Subjective patient satisfaction was higher in the mixed group [31]. The option that we suggest in this study which is the exchange of the MF-IOL with another MF-IOL, in our opinion, is the best approach to target the initial expectations of the patient of total spectacle independence for all distances.

This study is considered by our group as a pilot study, and thus, one of its most important limitations is the small number of patients included. In addition, different types of MF-IOLs were evaluated, but their outcomes according to the type of optical design could not be analyzed separately due to the insufficient sample size on each expected subgroup. Furthermore, this was a retrospective study. However, it shall be considered as a preliminary report where we suggest a new approach that can assist in alleviating patient’s dissatisfaction after MF-IOL implantation and without abandoning the initial patient’s target of being free of reading glasses. Another limitation is the lack of a control group with patients that had the MF-IOL exchanged by monofocal IOL. An ongoing study of our group analyzing patient’s satisfaction after MF IOL exchange by a monofocal IOL may allow us to compare such groups. Finally, another limitation of the study is the fact that part of it was based on subjective questionnaires answered by the patients. Hence, the study may have a certain amount of patient expectation bias.

As discussed earlier, neuroadaptation is the way in which our brain follows to adjust to any sensory input [7]. Everyone has a different capacity to neuroadapt to visual aberrations and this depends on many factors including age, state of mind, and fatigue. Although 80% of the patients with MF1 had binocular vision equal or more than 0.097 logMAR, patients still had subjective visual complaints. This is when neuroadaptation plays a major role and that is why CDVA is not the ideal outcome measured in such patients. This was confirmed by recent studies where patients with good vision complained of visual symptoms that decreased with time. This has been demonstrated by functional magnetic resonance imaging of patients implanted with MF-IOLs under grating stimuli [5]. In the early postoperative period, the increased activity of cortical areas represented the beginning of the neuroadaptation processes to MF-IOLs following a decrease in this activity six months postoperatively, suggesting long-term adaptation [6].

Therefore, adequate tools such as validated questionnaires should be used for the measurement of subjective outcomes [32]. In this study, QoV and visual function were assessed using the validated QoV and VF-14 questionnaires, respectively. There was a statistically significant improvement in the scores of both questionnaires, indicating a decrease in symptoms. In addition, the percentage of patients that would repeat the surgery increased from 13.3% with MF1 to 80% with MF2, taking into consideration that mostly the same types of lenses were used for MF1 and MF2 in different patients. This suggests that the same IOL may behave differently with diverse outcomes in different patients.

It was reported in some studies that blurred vision encountered by patients implanted with MF-IOLs can be due to residual postoperative ametropia, especially astigmatism [4, 33–35]. However, in our series, the patients with significant residual ametropia were tested concerning satisfaction with the use of glasses, with no success concerning their subjective symptoms. Furthermore, our findings showed that CDVA and subjective satisfaction increased significantly even when the spherical equivalent changed insignificantly. Therefore, the evidence raised in this investigation allow us to conclude that, in selected cases, the problem of MF-IOL neuroadaptation failure may be successfully managed with the exchange of the implanted MF-IOL by another with a different optical profile. Neuroadaptation is a very delicate and subjective process. As observed in this study, the same MF-IOL model can be accepted by one patient’s brain but be rejected by the brain of another patient and develop neuroadaptation failure. That is why at this point it is difficult to know definitively that exchanging one MF-IOL with another MF-IOL would give the same outcome and eventually solve the problem of neuroadaptation failure. It seems that different neural learning processes happen in the neuroadaptation process to different MF optical profiles. This hypothesis deserves further confirmation probably using already described brain function technologies to assess the brain processing of visual input associated with the new MF-IOL [5, 6].

## Conclusions

In conclusion, exchanging a MF-IOL for another MF-IOL with a different optical profile can offer a successful second opportunity to dissatisfied patients affected by neuroadaptation failure following MF-IOL implantation, both to eliminate their dissatisfaction and to keep the near visual advantages of MF-IOLs and preserve the patient’s expectations for far and near vision spectacle independence.

## Data Availability

Available upon request.
